# Intensified specimen collection to improve tuberculosis diagnosis in children from Rural South Africa, an observational study

**DOI:** 10.1186/1471-2334-14-11

**Published:** 2014-01-09

**Authors:** Tania A Thomas, Scott K Heysell, Prashini Moodley, Romualde Montreuil, Xia Ha, Gerald Friedland, Sheila A Bamber, Anthony P Moll, Neel Gandhi, William E Brant, Willem Sturm, Sarita Shah

**Affiliations:** 1 Division of Infectious Diseases & International Health, University of Virginia, PO Box 801337, Charlottesville, VA 22908-1337, USA; 2 University of KwaZulu-Natal, Durban, KZN, South Africa; 3 Department of Medicine, Albert Einstein College of Medicine, Bronx, NY, USA; 4 Yale University School of Medicine, New Haven, CT, USA; 5 Philanjalo, Tugela Ferry, KZN, South Africa; 6 Department of Radiology and Medical Imaging, University of Virginia, Charlottesville, VA, USA

**Keywords:** Tuberculosis, Diagnosis, Childhood, Drug resistance, South Africa

## Abstract

**Background:**

In drug-resistant TB settings, specimen collection is critical for drug-susceptibility testing (DST). This observational study included multiple specimen types collected from pediatric TB suspects with the aim to determine diagnostic yield and inform clinical practice in children with drug-resistant and drug-susceptible TB.

**Methods:**

From 03/2009-07/2010, TB suspects aged ≥6 months and ≤12 years were recruited among outpatient and inpatient settings. Subjects were new TB suspects or had persistent symptoms despite ≥2 months of TB treatment. The protocol included collection of a single blood and urine specimen, a single sputum induction and, if inpatients and <5 years of age, collection of 3 gastric aspirates (GA). Samples were cultured on solid and/or liquid media. DST was by 1% proportion method.

**Results:**

Among 118 children with possible, probable or confirmed TB, the mean age was 4.9 years [SD 3.2] and 64 (62%) of those tested were HIV-positive. Eight (7%) subjects were culture-positive from at least one specimen; yield did not differ by HIV status or TB treatment history. Among those with positive cultures, 7/8 (88%) were from induced sputum, 5/6 (83%) from GA, 3/8 (38%) from blood, and 3/7 (43%) from urine. In subjects with both induced sputum and GA collection, sputum provided one additional case compared to GA. Multidrug resistant (MDR)-TB was detected by urine culture alone in one child >5 years old. Pan-resistant extensively drug resistant (XDR)-TB was identified by cultures from all sites in one subject.

**Conclusions:**

TB was cultured from HIV-positive and -negative children, and allowed for identification of MDR and XDR-TB cases. Urine and induced sputum each provided an additional TB diagnosis and, when compared to GA, may be considered a less invasive, same-day method of specimen collection for childhood TB suspects. This study illustrates the continued challenges and limitations of available strategies for pediatric TB diagnostics.

## Background

Nearly 500,000 cases of tuberculosis (TB) are estimated to occur annually among children less than 15 years of age worldwide [1,2]. However, accurate measures of the true pediatric TB burden are hindered by challenges in confirming a diagnosis [3,4]. Clinical and radiological findings of pediatric TB are often less specific compared to those among adults. Additionally, smear microscopy and culture confirmation are limited by perceived difficulties in obtaining adequate specimens and the paucibacillary nature of pediatric TB; the reported culture yield is between 10-25% [5,6]. Difficulty in TB diagnosis is magnified in the HIV-infected child as the yield for diagnostic evaluation may be diminished by the other mimics of TB presentation, including opportunistic pulmonary infections, failure to thrive and HIV-associated lymphadenopathy.

The need for a confirmed diagnosis of TB is becoming increasingly important in the era of drug-resistant TB [7,8]. By age strata, children under 15 years of age bear the highest proportion of multidrug-resistant (MDR)-TB among populations outside of eastern and central Europe [9]. In settings with low TB prevalence, it may be possible to circumvent the need for culture-confirmation in a child provided that the drug-susceptibility pattern from the TB contact is known. However, in settings of high MDR-TB prevalence, this key information is often unknown and safe administration of first-line TB treatment without culture confirmation may be precluded. In the region surrounding our study site, the Umzinyathi district of KwaZulu-Natal, South Africa, drug-resistant TB is exceedingly common: approximately one in three people with culture confirmed TB have MDR-TB and one in six have extensively drug resistant (XDR)-TB [10]. Because nosocomial and community spread of drug-resistant TB strains have been documented among adults and children, knowledge of a patient’s drug-susceptibility pattern is essential to provide optimal treatment [11,12]. Drug-susceptibility testing for TB can be achieved via mycobacterial culture or newer nucleic acid amplification tests; however, the optimal performance of all of these techniques still depends on adequate specimen collection [13,14].

Systematic evaluation of pediatric sampling methods is an important area of study to maximize chances of achieving a pathogen-based diagnosis efficiently. Historically, gastric aspiration after overnight fasting has been the accepted method of specimen collection from young children to evaluate for TB disease. However, the need for procedural skills and overnight hospitalization often preclude the use of this invasive test in low resource areas where the burden of TB may be greatest [15]. In recent years, less invasive methods of collecting respiratory tract specimens, including sputum induction and nasopharyngeal aspiration, have been evaluated in the context of research settings [5,16]. Results suggest at least equivalent yield from induced sputa [17,18] and nasopharyngeal aspirates [19] compared to gastric aspirates. Among pediatric inpatients from a tertiary center in Cape Town, South Africa, the culture yield from a single induced sputum specimen was equivalent to three consecutive gastric aspirates [5]. Among pediatric TB suspects attending a community clinic in Cape Town, positive yield from sputum induction led to TB treatment initiation in 21.6% additional children compared to clinical diagnosis [6]. However, the performance of sputum induction has not been validated in a rural setting under routine clinical conditions, despite the potential use as a same-day method of obtaining quality respiratory specimens.

Other specimens, including blood and urine, have not been thoroughly evaluated for mycobacterial culture from children. These bodily fluids are more easily obtainable than gastric contents and may offer a unique opportunity for detection among young children, particularly those with HIV co-infection, who are more likely to have disseminated forms of TB. Recently, we have found blood cultures to provide an accessible means of diagnosing drug-susceptible, MDR- and XDR-TB in adults co-infected with HIV [20]. Therefore, the use of blood and urine cultures in populations of children with a high prevalence of HIV may be of additive yield, particularly when respiratory specimens are difficult to obtain [20-22]. In this study, we aimed to determine the diagnostic yield of routine sputum, gastric aspirates, blood and urine cultures among pediatric TB suspects at a rural hospital in South Africa and inform clinical practice.

## Methods

### Setting

This study was conducted in the rural town of Tugela Ferry, South Africa. The provincial incidence of TB is estimated at 1,100 per 100,000 population annually, with approximately 11% occurring in children under 10 years [23]. Approximately 80% of adult and pediatric TB cases are HIV co-infected [23].

Patients were enrolled from a 355-bed district government hospital and affiliated on-site ambulatory departments consisting of outpatient and casualty departments as well as an HIV Clinic, all of which serve children. Relevant inpatient facilities include a 45-bed pediatric ward.

### Study design

We conducted an observational study among children ≥6 months to ≤12 years old enrolled between March 2009 and July 2010. Children suspected to have tuberculosis were referred to the study by hospital physicians. Patients were eligible if they had at least one of the following: chronic cough, failure to improve after pneumonia treatment, contact with a TB case, failure to thrive, painless superficial lymphadenopathy, signs of meningitis which were not responsive to antibiotics, or chest radiograph suggestive of TB. Patients were excluded if they were on anti-TB treatment for >2 days, or recently defaulted on TB treatment. In addition, all children who failed to improve clinically despite ≥2 months of TB treatment were eligible for enrollment to enrich sampling of children who may have drug-resistant TB.

### Study procedures

Aside from the intensified specimen collection, the evaluation for tuberculosis was performed per routine practice as per the hospital physicians. Research interventions included structured interviews to obtain demographics, medical/family histories, and TB exposures; prior history of TB was verified by searching through hospital DOTS registers, TB treatment cards and medical charts. Results of routine clinical investigations including anthropometrics, hemoglobin, albumin, erythrocyte sedimentation rate, HIV antibody reactivity, and CD4 percentage (when applicable) were abstracted from medical charts. Tuberculin skin testing was not routinely conducted during the study period. Chest radiographs were obtained per standard practice and included predominantly single AP views which were digitized and systematically interpreted by a study radiologist (WEB) who was blinded to the patients’ clinical and microbiological results.

As part of the intensified specimen collection protocol, all participants were to have blood, urine, and sputum collected. Venous blood (1-5 mLs) was directly inoculated into a Bactec MycoF-lytic bottle (Becton Dickinson, Sparks, MD, USA), which was stored at room temperature until incubated in the Bactec automated blood culture system, typically within 24 hours. Urine samples were obtained as first-morning, clean-catch specimens from children able to spontaneously void, and for younger children via a sterile bag secured to the perineum after skin disinfection with alcohol. A minimum of 5 mL of urine was collected and stored at 4°C until processed as detailed below.

Inpatients under 5 years of age had three consecutive morning gastric aspirates collected after an overnight fast as per standard protocol [1]. All children underwent a single sputum induction by the hospital’s trained respiratory physiotherapists following a standard protocol [1]. Of note, since 2008, the hospital physiotherapy department has been performing sputum induction routinely on adult and pediatric patients in an open-air setting separated from patient areas with personal protective equipment provided for parents and staff. Gastric aspirates were performed prior to sputum induction.

Specimens were transported daily to the TB laboratory at the University of KwaZulu-Natal in Durban where microscopy, culture, and drug-susceptibility testing was performed. Sputum, gastric aspirate, and urine specimens were digested and decontaminated by the N-acetyl-L-cysteine (NALC)-NaOH method. An aliquot was used for fluorescent microscopic sputum examination for acid-fast bacilli (AFB; auramine-stained) and the remainder was split for parallel culture on Middlebrook 7H11 agar plates and automated broth culture via the Bactec MGIT-960 system. Solid cultures were monitored at 21- and 42-days; liquid cultures were monitored continuously for 42 days. A negative culture was one without growth in either system after 42 days. For all positive mycobacterial cultures, biochemical testing with niacin accumulation and nitrate reduction was used to confirm the species as *Mycobacterium tuberculosis*.

Drug-susceptibility testing was performed on all *M. tuberculosis* isolates using the 1% proportional method on Middlebrook agar with standard critical concentrations for isoniazid, rifampin, streptomycin, ethambutol, ofloxacin, kanamycin, capreomycin, ethionamide and niacinamide [24]. MDR-TB was defined as resistance to isoniazid and rifampin, while XDR-TB included further resistance to ofloxacin and at least one injectable agent (kanamycin or capreomycin). Niacinamide was used as a proxy for pyrazinamide, and if resistant, confirmatory testing was performed including evaluation for pyrazinamidase production and mutations in the *pncA* gene. If non-tuberculous mycobacteria were identified, further speciation or drug-susceptibility testing was not pursued.

The remainder of clinical management, including the need for any TB treatment, was directed by the child’s medical doctor according to South African National TB Control Programme Guidelines [25]. Typically, children from this region on TB treatment are monitored by the DOTS Programme nurses, however participants in this study were asked to return for a follow-up visit with a study-related pediatrician two months after enrollment to monitor for symptom resolution and determine final diagnostic classification.

### Definitions

Patients were retrospectively categorized as having possible, probable, or confirmed TB based on the World Health Organization (WHO) guidelines [26]. Possible TB was defined as having a cough for ≥14 days and at least two of the following symptoms: fever, night sweats, weight loss, failure to thrive, or painless lymphadenopathy. Probable TB patients included those who met criteria for Possible TB and had two of the following in addition: known TB contact, chest radiograph consistent with TB, or favorable response to TB treatment after 2 months. Confirmed TB was defined as having any culture-positive specimen for *M. tuberculosis*.

The nutritional status of the children was determined by calculating height-for-age z scores (HAZ) and weight-for-age z-scores (WAZ) (AnthroPlus, v1.0.3, WHO). Children with a Z-score ≤ -2 standard deviations (SD) below the mean were defined as being malnourished, and those ≤ -3 SD were severely malnourished.

### Statistical analysis

We described simple frequencies for exposure variables, stratified by TB classification (possible, probable or confirmed). To determine risk factors associated with probable or confirmed TB, we combined participants with probable and confirmed TB and compared them to those with possible TB. Chi-square tests were used for dichotomized variables; t-test and Wilcoxon rank-sum tests were used for continuous variables. All analyses were performed using SAS Statistics 9.2 software (SAS Institute, Cary, North Carolina, USA).

### Ethical considerations

The study was approved by the ethics committees of University of KwaZulu-Natal, Albert Einstein College of Medicine, and Yale University. Permission was also granted by the KwaZulu-Natal Department of Health. Written permission from the parent/guardian to participate in the study, and verbal child assent when applicable, was obtained from all participants.

## Results

**Figure 1:**
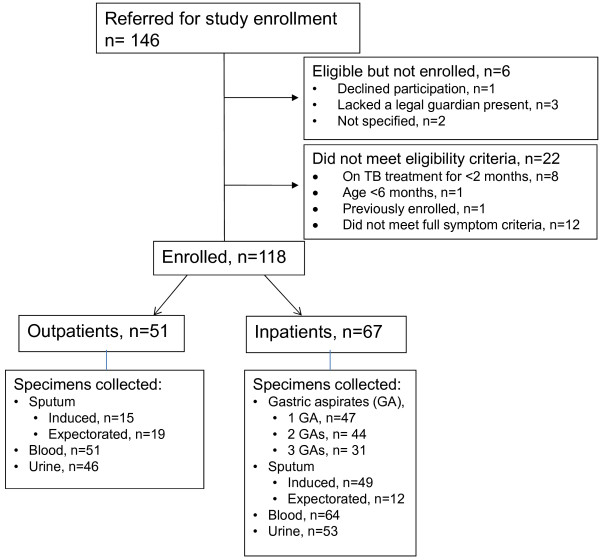
Flow diagram of enrollment.

A total of 118 pediatric TB suspects met enrollment criteria (Figure 1). There were 85 (72%) children categorized as having possible TB, 25 (21%) as probable TB, and 8 (7%) had culture-confirmed TB. The mean age was 4.7 years. Demographic and clinical characteristics are reported in Table 1. Reflective of the high prevalence of HIV in the community, 59 (50%) children had at least one HIV-infected member of the household, while 37 (31%) patients were orphaned by at least one parent.

**Table 1:** Demographic & clinical information about pediatric TB suspects, N = 118

	**Total**	**Possible TB**	**Probable & Culture-confirmed TB**	**p-value**
	**n = 118, (%)**	**n = 85, (%)**	**n = 33, (%)**	
Age, mean years [SD]	4.7 [3.1]	4.9 [3.4]	4.4 [3.2]	NS
Female gender, n (%)	53 (45)	39 (46)	14 (42)	NS
Mother as primary caregiver	70 (59)	50 (59)	20 (61)	NS
Either parent deceased	37 (31)	26 (31)	11 (33)	NS
HIV-infected mother	50 (42)	35 (41)	15 (45)	NS
Other HIV-infected family member	9 (8)	7 (8)	2 (6)	NS
TB contact reported	62 (53)	37 (44)	25 (76)	0.001
Drug-resistant TB contact reported	4 (3)	3 (3)	1 (3)	NS
Contact with “chronic cougher”	19 (16)	13 (15)	6 (18)	NS
Prior TB	37 (31)	28 (33)	9 (28)	NS
Prior hospitalization (≥1)	31 (26)	24 (28)	7 (21)	NS
Malnourished	52 (44)	34 (40)	18 (55)	NS
Severely malnourished (n, % of malnourished)	42 (81)	26 (76)	16 (89)	0.09
Tested for HIV	99 (84)	73 (86)	26 (79)	NS
HIV positive	64 (65)	47 (64)	17 (65)	NS
On HAART, n (% of HIV+)	32 (50)	25 (53)	7 (41)	NS
CD4%, median [IQR]	16.8 [11.5-22.3]	16.7 [13.7-22.3]	17 [7.28-19.2]	NS
Hemoglobin, median g/dL [IQR]	9.9 [8.8-11.2]	10.3 [9.2-11.2]	8.9 [7.3-10.8]	0.04
Albumin, median g/L [IQR]	32.0 [28.1-37.0]	33.9 [29.0-37.0]	28.8 [25.0-30.8]	NS
Erythrocyte Sedimentation Rate, median [IQR]	32.0 [14.0-75.0]	30.0 [14.0-75.0]	36.0 [13.5-75.0]	NS
Abnormal chest radiograph consistent with TB	84* (85)	55* (79)	29* (100)	0.03
Enrolled while failing ≥2 mo TB treatment	8 (7)	6 (7)	2 (6)	NS
Started on TB treatment	59 (50)	37 (44)	22 (67)	NS

*Of 99 children with chest radiographs available for review.

Children categorized as probable/confirmed TB were more likely to report a known TB contact (p = 0.001), which is likely reflective of the criteria used for the case definition. Four (3%) children reported contact with a drug-resistant TB case: none were confirmed to have drug-resistant TB. The overall prevalence of malnutrition was high (44%); among malnourished children, severe malnutrition was found in 42 (81%). Among children tested for HIV, 64 (65%) were HIV-positive, of which 32 (50%) were receiving anti-retroviral therapy (Table 1).

Laboratory results did not differ significantly between groups, aside from median hemoglobin level, which was lower among those categorized with probable/culture confirmed disease (p = 0.04). Of the 99 (84%) subjects with a chest radiograph available for review, all 29 (100%) children categorized with probable/culture-confirmed disease had findings suggestive of TB (p = 0.03), which may be reflective of the case definition criteria. The 8 (7%) children enrolled while clinically failing to respond to ≥2 months of TB treatment were more likely to have probable TB.

The per-patient culture yield for *M. tuberculosis* was 7% (n = 8). The contribution of each of the diagnostic methods is shown in Table 2. Smear-positive disease was seen in 50% of culture-positive cases. Among the five children who were culture-positive by sequential gastric aspirates, the yield from the first gastric aspirate was equivalent to multiple collections. Induced sputum specimens from these five children were also culture-positive. Sputum induction was well tolerated overall: no child experienced prolonged respiratory distress, one child required brief supplemental oxygen and two had post-procedural vomiting. Respiratory physiotherapists reported ease with the procedure. However, there were 23 (19%) children who had unsuccessful collection of respiratory specimens due to dry cough/insufficient specimen volume and scheduling limitations between patients and physiotherapists.

**Table 2:** **Comparison of sample collection yield among patients culture-positive for *M. tuberculosis *** **by any collection method**

TB status*	**Sputum smear**	**Sputum culture**	**Gastric smear**	Gastric culture**	**Blood**	**Urine**	DST interpretation***	**Age (years)**	**HIV status**	**CD4%**
Possible	-	+	-	+	-	-	DS	1.3	Negative	n/a
Possible	-	+	+	+	-	-	DS	2.2	Negative	n/a
Possible	-	+	-	-	-	-	DS	1.7	Positive	5.3
Possible	-	-	NC	NC	-	+	MDR	8.3	Positive	17.4
Probable	-	+	-	+	-	NC	DS	2.0	Negative	n/a
Probable	-	+	+	+	+	-	DS	1.5	Negative	n/a
Probable	+	+	NC	NC	+	+	DS	7.0	Positive	2.6
Probable	+	+	+	+	+	+	XDR	11	Positive	10.9
**Total positive (% of collected)**	2 (25)	7 (88)	3 (50)	5 (83)	3 (38)	3 (43)				

*TB status prior to obtaining microbiologic confirmation.

**Among the subjects who had positive cultures by gastric aspirates, 4 (80%) demonstrated culture-positivity from all three aspirates while 1 (20%) child only demonstrated culture-positivity from the first two aspirates and left the hospital prior to collection of the third aspirate.

***DST: drug susceptibility testing.

XDR: extensively drug-resistant tuberculosis. DS: drug susceptible. MDR: multi-drug resistant. NC: not collected.

n/a: not applicable.

One patient was *M. tuberculosis* culture-positive by the urine specimen only, despite presenting with pulmonary symptoms. While blood cultures were positive in three culture-confirmed subjects (two who were HIV-positive), there was no additive yield above sputum. Of potential alternative diagnoses, cultures did yield non-tuberculous mycobacteria from the blood (n = 2) and urine (n = 1) when *M. tuberculosis* was not found. This study did not include any other testing to confirm non-TB diagnoses.

Among the eight children with culture-confirmed TB, two were found to have drug-resistant TB; both were co-infected with HIV and demonstrated excellent virologic response to antiretroviral medication. One child with MDR-TB (CD4% = 17.4) was diagnosed by urine culture only; one child (CD4% = 10.9) was septic with XDR-TB [27]. Neither of the two children was known to have contact with a drug-resistant TB case. The child with XDR-TB was infected with an isolate that was resistant to all nine drugs tested, including those that make up the backbone of the provincial standard XDR-TB treatment regimen. Five culture-confirmed cases were found to be niacinamide resistant, but follow-up testing revealed all to have pyrazinamidase activity and none had *pncA* mutations identified.

Of all participants, 59 (50%) were started on TB treatment – including 8 (13%) who were enrolled while being on TB treatment for ≥2 months, 44 (75%) who were started after enrollment, and 7 (12%) who initiated treatment after the two-month follow-up visit. Seven (6%) subjects had died within two months of enrollment into the study: three subjects were possible TB cases, two had probable TB, and two had culture-confirmed disease including one child with XDR-TB. The majority, n = 6 (86%), were on TB treatment at the time of death. Eighty-six percent were also HIV-positive.

## Discussion

Diagnosis of pediatric TB remains a global public health and clinical challenge, given the limitations of current tests and the paucibacillary nature of disease. As the incidence of drug-resistant TB increases, diagnostic confirmation and drug-susceptibility testing becomes more important for appropriate management. In this study, we examined the diagnostic yield of multiple specimen types for pediatric TB in a rural, resource-limited operational setting. We found that, despite intensive specimen collection, culture confirmation of *M. tuberculosis* remained low overall and extrapulmonary specimens did not provide additive culture yield over respiratory specimens. However, culture confirmation allowed for identification of drug-resistant TB in two of eight culture-positive cases, including one case of XDR-TB resistant to all nine antibiotics tested and one case of MDR-TB in whom the diagnosis was only confirmed via the urine specimen. Additionally, the diagnostic yield from same-day, outpatient sputum induction was equivalent to that of three, inpatient gastric aspirates and may provide a practical alternative to specimen collection in resource-limited settings.

Clinicians are often dependent upon a clinical means of diagnosis for pediatric TB suspects. However, the WHO classification schema used in this study for possible and probable TB cases was not able to accurately categorize our participants who were found to have culture-confirmed disease. In this cohort, 4 of the 8 children who had culture-confirmed TB only met symptom criteria for “possible TB”, emphasizing the limited sensitivity of this schema. Clinical findings can be non-specific especially in areas, such as ours, with a high prevalence of HIV and/or malnutrition, each of which may confound the presentation of other opportunistic infections that resemble TB. Clinical scoring systems have been used throughout the world with varying degrees of success, especially when applied to HIV-prevalent settings [28-31]. Newer clinical consensus guidelines for pediatric pulmonary TB classification have been published, [32] which holds promise for improving classification. But an acceptable and feasible gold standard diagnostic test for children remains elusive.

Abnormal findings by chest radiograph were associated with probable/culture-confirmed disease in this cohort. Radiologic findings can often provide objective data to support a diagnosis of TB disease [33]. However, given the spectrum of radiographic manifestations based on age, disease stage and severity, and host immune competence, the specificity may be low and expertise at pediatric radiography is required locally, which may not be routinely available in rural settings. The ongoing installation of digital radiographic units in resource-limited regions will allow for rapid consultation with radiologists experienced in pediatric radiology by internet or cell-phone data transmission [34]. Until improved diagnostic capabilities are available, emphasis should be placed on accurate classification of pediatric chest radiographs at the local level as a helpful diagnostic aid [35].

The diagnosis of drug-resistant TB cannot accurately be made on clinical grounds alone and requires an adequate specimen for culture or molecular testing. In this high drug-resistant TB and HIV prevalence setting, we found two confirmed drug-resistant TB cases out of the eight culture-positive cases in total; both drug-resistant cases were among HIV-co-infected children. Importantly, neither child was known to have contact with a drug-resistant TB case, so would not have been identified as “high-risk” by epidemiologic exposure history. Increasing data have shown that treatment of pediatric MDR-TB [36,37] and XDR-TB [12], even in high HIV prevalence settings, has excellent outcomes. In addition, transmission of TB from children has been demonstrated [38,39]. Thus, accurate diagnosis of MDR- and XDR-TB in children has great potential to both save lives and reduce transmission. But, the low yield from current diagnostic tests remains a challenge.

An important finding from this study is the yield and feasibility of sputum induction performed at this rural district-level hospital under routine clinical conditions. Here, sputum induction was tolerated without significant adverse effects and provided equivalent yield to multiple gastric aspirates, consistent with studies in referral centers in South Africa [5]. Sputum induction is an attractive diagnostic alternative for resource-limited settings as it offers practical advantages of a same-day procedure, while also providing an adequate specimen for culture or molecular-based diagnostics [14]. While HIV status did not seem to affect culture-positivity, our small sample size limits our ability to detect whether HIV status or other risk factors truly affected the yield from extra-pulmonary specimens. Additionally, a subset of patients enrolled from the outpatient setting were not able to successfully undergo the procedure.

The culture yield in our study was lower than expected from this TB-endemic region, though falls within the range of published studies from settings with high HIV prevalence [14,15,40]. In comparison to other published studies, our low yield may be explained by the rural district setting of this study site compared to urban referral centers, as the latter may see a higher proportion of TB suspects with later-stage presentations and higher bacillary burden. A proportion of children did not have all specimens collected for various reasons including: inadequate fasting for sputum induction, in adequate time available for sputum induction, dry cough/insufficient specimen volume, inability to produce urine during the outpatient encounter, and unsuccessful phlebotomy/insufficient specimen volume. Additionally, our yield may have been affected by the lower specificity of clinical diagnoses and the presence of other pulmonary infections mimicking TB in this cohort with a high prevalence of HIV and severe malnutrition. The broad eligibility criteria may have played a role in the low culture rate as evidenced by only 28% of subjects who met criteria for probable/confirmed TB and the relatively low proportion of children overall who were deemed to require TB treatment based on clinical criteria. As a result, the low culture yield limited our ability to identify risk factors for confirmed TB disease in this study. Overall, these limitations hindered our ability to draw definitive conclusions and further, larger prospective studies are needed.

## Conclusions

Although greater attention to TB diagnosis in recent years has led to landmark gains, the majority of this benefit has been realized in smear-positive patients [41,42]. Since children are known to have paucibacillary disease, diagnostics that focus on the challenges of specimen collection and unique disease presentation are urgently needed [43]. Owing in part to these challenges, the current burden of pediatric drug-resistant TB remains unknown, thereby limiting efforts to advocate for improved diagnostics and drug formulations for children. It is estimated that only 5% of adult MDR TB cases are diagnosed [44,45]. It is further sobering to appreciate that even fewer of the estimated 40,000 cases of pediatric MDR TB are diagnosed. The undiagnosed and unrecognized epidemic of pediatric TB is being borne silently by children worldwide. Ending childhood deaths due to TB requires strengthening of TB resources and systems as well as new diagnostics and treatments tailored to the special requirements of children at risk.

## Abbreviations

AFB: Acid-fast bacilli; DOTS: Directly-observed therapy short course; DST: Drug susceptibility testing; GA: Gastric aspirates; HAZ: Height-for-age Z score; HIV: Human immunodeficiency virus; MDR: Multidrug resistant; SD: Standard deviation; TB: Tuberculosis; WAZ: Weight-for-age Z score; WHO: World Health Organization; XDR: Extensively drug resistant.

## Competing interests

The authors declare that they have no competing interests.

## Authors’ contributions

TAT, SKH, GF and SS designed and supervised the study. SAB and AMP contributed to the supervision and conduct of the study. PM and WS supervised the laboratory procedures. RM participated in data collection. XA performed statistical analysis of the data. WEB analyzed and interpreted the radiographic data. TAT, SKH, RM, XA and SS made contributions in analyzing and interpreting the data. TAT, SKH, RM, and SS wrote the manuscript. All authors revised the manuscript critically for important intellectual content. All authors read and approved the final manuscript.

## Pre-publication history

The pre-publication history for this paper can be accessed here:

http://www.biomedcentral.com/1471-2334/14/11/prepub
